# Gram-positive enhancer matrix delivering FVpE via M cell targeting elicit protective mucosal and adaptive immune responses against *Helicobacter pylori* infection

**DOI:** 10.3389/fimmu.2025.1697591

**Published:** 2025-11-04

**Authors:** Furui Zhang, Yaqin He, Hongpeng Liu, Jing Wu, Xin Li, Jiale Chen, Linhan Ni, Zhen Zhang, Juan Chen, Kunmei Liu, Le Guo

**Affiliations:** ^1^ School of Laboratory, Ningxia Medical University, Yinchuan, China; ^2^ Ningxia Key Laboratory of Clinical and Pathogenic Microbiology, General Hospital of Ningxia Medical University (the First Clinical Medical College of Ningxia Medical University), Yinchuan, China; ^3^ Laboratory, Xi’an Hospital of Traditional Chinese Medicine, Xi’an, China; ^4^ College of Pharmacy, Ningxia Medical University, Yinchuan, China; ^5^ Department of Geriatrics and Special Needs Medicine, General Hospital of Ningxia Medical University, Yinchuan, China; ^6^ Department of Pulmonary and Critical Care Medcine, General Hospital of Ningxia Medical University, Yinchuan, China; ^7^ Ningxia Key Laboratory of Cerebrocranial Diseases, Ningxia Medical University, Yinchuan, China

**Keywords:** gram-positive enhancer matrix, *H. pylori*, M cells, mucosal immune, adaptive immune

## Abstract

**Background:**

The Gram-positive enhancer matrix (GEM) is a novel mucosal vaccine delivery system based on lactic acid bacteria (LAB). *Helicobacter pylori* (*H. pylori*) mainly colonize the gastric mucosa and thus induce various gastric diseases. Hence, the development of an efficient mucosal vaccine is expected to be a new strategy for the prevention and treatment of *H. pylori*.

**Methods and Results:**

This study is based on the GEM delivery system, which constructs an oral vaccine targeting intestinal M cells, GEM-SAM-FVpE. Here, SAM represents the surface anchoring protein (cA) and the M cell-targeting peptide (Mtp), thereby enabling both efficient display on the GEM particle and targeted to intestinal M cells. And FVpE denotes the *H. pylori* multi-epitope antigen. As a results, GEM is able to successfully display the purified antigen SAM-FVpE on the surface, with a display efficiency of 90%. Meanwhile, GEM-SAM-FVpE enhances antigen presentation efficiency and activates DCs by upregulating MHC II and costimulatory molecules (CD80/CD86/CD40), and increasing the secretion of related cytokines. *In vivo* experiments indicate that oral administration of the GEM-SAM-FVpE significantly induces the production of high titers of sIgA, serum IgG, and its subtype, initiating mucosal and humoral immune responses, and inhibiting the adhesion of *H. pylori* to normal gastric mucosal epithelial cells. In addition, by significantly activating Th1, Th2, and Th17, it initiates antigen-specific cellular immune responses. Finally, *H. pylori*-infected mice treated with GEM-SAM-FVpE can significantly reduce the colonization of *H. pylori* in gastric tissue while also decreasing gastric mucosal damage.

**Conclusion:**

GEM-SAM-FVpE can effectively induce protective mucosal responses and adaptive immune responses against *H. pylori* infection, providing a new scheme for the development of oral vaccines against *H. pylori*.

## 1 Introduction

*Helicobacter pylori* (*H. pylori*) is a gram-negative bacterium that selectively colonizes the gastric epithelium, surviving the stomach’s highly acidic environment primarily through urease production (1). Genetic analyses reveal that humans have coexisted with *H. pylori* for over 58, 000 years (2). The high prevalence of *H. pylori* infection is a major contributor to the global incidence of gastric cancer (3), with an estimated 75% of cases worldwide attributed to this pathogen (4). *H. pylori* was recognized in 1994 as a class I carcinogen by the International Agency for Research on Cancer (IARC) (5). With the growing body of infection on *H. pylori*, the scope, dosage, and duration of antibiotic use have significantly increased. Currently, the preferred treatment for *H. pylori* infection is bismuth quadruple therapy, which combines a proton pump inhibitor PPI, bismuth, and two antibiotics (6, 7). However, this regimen is associated with high costs, prolonged treatment durations, poor patient compliance, and an increased risk of antibiotic resistance during therapy. Given these limitations, there is an urgent need to develop novel therapeutic strategies for the effective prevention and treatment of *H. pylori* infections. Vaccines offer a promising approach for the prevention and eradication of *H. pylori*.

Recent advancements in *H. pylori* vaccine research include whole bacterial inactivated vaccines, subunit vaccines, and vector-based vaccines. For example, Suganya K. et al. evaluated the efficacy of a heat-inactivated *H. pylori* vaccine formulated with an aluminum phosphate adjuvant in mice. The study demonstrated a significant increase in IgG titers on days 21 and 28 post-immunization (8). Given that the virulence factors of *H. pylori* exhibit strong antigenicity and effectively elicit immune responses (9), developing subunit vaccines against *H. pylori* using genetic engineering technology has become a primary focus of research. So, the research developed a subunit vaccine, which was conducted a phase III clinical trial to evaluate the vaccine’s efficacy in *H. pylori*-negative children. The immune response rates were 72% and 65% at one year and three years of follow-up, respectively (10). Additionally, vector-based vaccines offer a unique advantage by delivering proteins directly to the site of action while avoiding degradation by gastric acid. Current studies primarily focus on bacterial vector vaccines, viral vector vaccines, and nanocarrier vaccines. However, the *H. pylori*’s ability to colonize the gastric mucosa and evade host immune responses significantly limits the efficacy of traditional injectable vaccines (9). Moreover, gastrointestinal vaccination poses challenges due to the stringent requirements for effective immune adjuvants and delivery systems.

A novel Lactic acid bacteria (LAB) surface presentation technique based on Gram-positive enhancer matrix (GEM) particles were utilized to delivery antigen (11). Such particles are derived from freshly grown LAB by thermal acid, which removes intracellular and extracellular macromolecules such as DNA, most bacterial proteins, and lipo-phosphatidic acids, but leaves an intact peptidoglycan (PGN) envelope (12, 13). Postbiotics, mixtures of non-viable microbial cells and their components, are noted for their safety. GEM are engineered postbiotics that go beyond simply retaining the bacterial structure to act as a targeted delivery platform (14). The GEM surface display system has the following advantages as a delivery vehicle for vaccines: firstly, it is safe, convenient and stable; in addition, it has an auto-adjuvant effect (16); secondly, it has a high efficiency of mucosal delivery: as the GEM particles maintain their original size and structure of about 1 μM, they can adhere to mucosal surfaces or selectively to M cells, which can efficiently stimulate innate responses and evoke adaptive immune responses against pathogens (17). Because of the unique advantages of high safety and efficiency of GEM, mucosal vaccines against a variety of respiratory and gastrointestinal infectious diseases have been successfully prepared, and all of them have shown good immune-protective effects (18–20). The key element that enables antigens to be delivered by GEM is the anchoring protein (cA). cA refers to the C-terminal domain of AcmA, the main autolysin of *L.lactis*, and can specifically bind GEM. By fusing cA with bacterial antigens and then mixing them with GEM, the heterologous bacterial antigens can be displayed on the surface of GEM through non-covalent bonds under the action of cA at room temperature, thereby facilitating the delivery of bacterial antigens (15).

M cells are specialized cells found in the intestinal tract’s mucosal epithelium. They are in charge of absorbing and delivering antigens to immune tissues in the submucosal layer, which starts both systemic and local immune reactions. On their surface, M cells contain a number of distinct receptors (integrins β1, GP2) that are effective at identifying and absorbing antigens (21). The immunogenicity of the vaccine can be increased by producing bacterial antigens in combination with ligands that target M cells, which would greatly increase the antigens’ absorption efficiency on the mucosal surface (22). Mucosal immunity is the first line of defense against pathogen invasion, and vaccines that target M cells can deliver antigens directly to mucosa-associated lymphoid tissues (MALT) to induce local sIgA production, forming an effective immune barrier at the early stage of infection to prevent colonization and spread of pathogens (23). In addition to triggering local mucosal immunity, M cells can also send antigens to T and B cells through antigen-presenting cells, which starts systemic humoral immunity (24). This dual immune activation mechanism provides a more comprehensive protective effect for the vaccine. Furthermore, oral vaccinations can be disrupted by the gastrointestinal environment, and traditional vaccines have trouble successfully triggering mucosal immunity. Targeting M cells increases the vaccine’s durability and addresses the shortcomings of conventional vaccinations in mucosal immune activation by delivering antigen directly to the immunologically active region by oral delivery. Since *H. pylori* primarily colonizes the gastric mucosa, the M-cell-targeted vaccination is more efficient at getting rid of *H. pylori* and reducing the development of associated illnesses because it may trigger the immune response of the gastric mucosa and operate directly on the infection site (25).

**Scheme 1:**
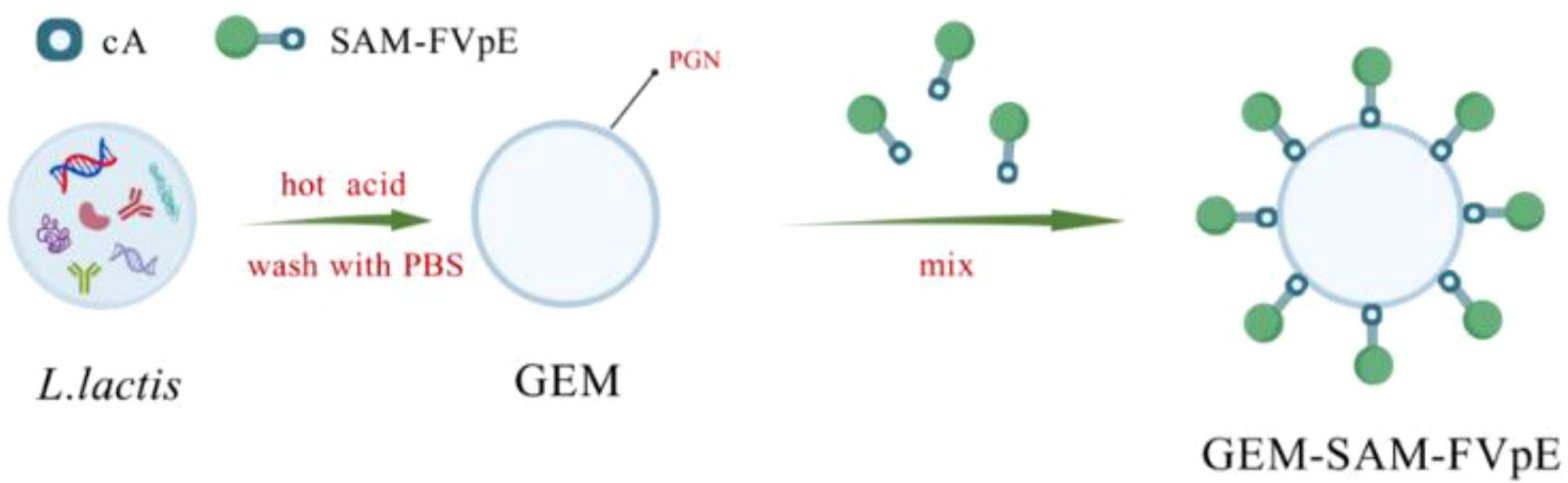
*L. lactis* was subjected to heat and acid treatment to obtain GEM. Under the cA, the M cell-targeting *H. pylori* multi-epitope vaccine was bound to the surface of GEM, forming the particles vaccine GEM-SAM-FVpE.

Accordingly, the present study was based on the GEM particles mucosal vaccine delivery system for the development of M-cell-targeted *H. pylori* vaccine—GEM-SAM-FVpE (**Scheme 1**). Determining the optimal amount of GEM binding to antigen by optimizing conditions, the M-cell-targeted *H. pylori* multi-epitope antigen (SAM-FVpE) was displayed on GEM particles to evaluate the targeting and immune activation effects of the particulate vaccine by mimicking the pathogens’ mechanism of activation of the body’s mucosal immune system.

## 2 Materials and methods

### 2.1 Strain and culture conditions

Both *Escherichia coli* DE3 and *Lactococcus lactis* NZ9000 were stored in the Key Laboratory of Pathogenic Microbiology, Ningxia Medical University. DE3 was routinely cultured at 37°C in LB medium, NZ9000 was expanded at 30°C in GM17 medium containing 0.5% glucose, and *H. pylori* was cultured on Colombia blood plates containing *H. pylori* bacteriostatic agent for 72 h at 37°C, 5% O_2_, 85% N_2_, 10% CO_2_, and 95% humidity.

### 2.2 Mice

5–6 weeks old BALB/c mice were purchased from Beijing Huafukang Biotechnology Co Ltd, kept in the SPF animal experiment center, and provided with sufficient food and water throughout the study period. The animal protocols were approved by the Animal Experiment Ethics Committee of Ningxia Medical University.

### 2.3 Preparation and identification of GEM-SAM-FVpE vaccine

NZ9000 was activated, amplificated at 150 rpm at 30°C, washed once with sterile distilled water, and resuspended with 10% volume of 0.1 M HCL in a boiling water bath for 30 min. Next, wash three times with sterile PBS to remove nucleic acids and proteins. After that, resuspend with sterile PBS, adjust its concentration to 1 U (1×10^8^ particles/mL). SDS-PAGE, transmission electron microscopy (TEM) and particle size analysis were used to evaluate the morphology and preparation effect of GEM. For fusion protein SAM-FVpE acquisition, we first designed the core element SAM containing cA (loading of fusion proteins on the GEM surface) and Mtp (targeted binding to M cells). Then, plasmids pCzn1-SAM-FVpE obtained by integrating SAM with *H. pylori* multi-epitope gene FVpE, which was introduced into the *E. coli* DE3, and the IPTG was added to the bacterial solution at 37°C for 4 h. The fusion protein SAM-FVpE was purified by Ni-NTA column affinity chromatography, and the purity and concentration of the fusion protein were analyzed. Then the optimal binding ratio between GEM and SAM-FVpE antigen analyzed by SDS-PAGE and Western blot. After that, the 1U GEM and 100 μg SAM-FVpE were mixed at room temperature for 30 min, the unbound protein was washed to obtain the GEM-SAM-FVpE vaccine. To assess the binding capability of SAM-FVpE to GEM, 200 μL sample was initially examined using TEM. Subsequently, the sample was diluted to an optimal concentration for further analysis by SDS-PAGE, flow cytometry, and immunofluorescence. Specifically, GEM and GEM-SAM-FVpE were blocked with 3% BSA-PBS for 30 min. After washing twice with PBS, the diluted samples were incubated with FITC-labeled anti-6*His antibody (1:100) for 2 h at room temperature. Then the fluorescence was observed after washing.

### 2.4 Construction of M-cell model and targeted analysis of GEM-SAM-FVpE vaccine

According to the method of Kerneis et al (26, 27), the M-cell model was constructed using the co-culture of Caco-2 cells and Raji B cells. Firstly, the effects of different amounts of GEM-SAM-FVpE on the viability of Caco-2 and Raji B cells were evaluated by CCK-8 assay. Caco-2 cells were cultured on the apical of 3 μm Transwell for 14 days, Raji B cells were added to the basolateral for 5–6 days (co-culture), and no Raji B cells (mono-culture) were set as a control. To verify the M cell model, the Transwell membrane was observed by scanning electron microscopy (SEM, S-3400N, Japan) on 19 days. In addition, the apical and basolateral media in mono-culture and co-culture on day 20 were collected separately to analyze alkaline phosphatase (ALP) activity by ALP detection kit (Beyotime, China).

After the model was successfully constructed, the M cell targeting of GEM-SAM-FVpE was verified. The AlexFluor^®^488-Anti His was used to label the GEM-SAM-FVpE in advance, then added to mono-culture and co-culture for 6 h, washed and fixed, observed by fluorescence microscopy (OLYMPUS, Japan). In addition, using ileal closed-loop tests, the M cell targeting of GEM-SAM-FVpE was confirmed *in vivo*. Following the mice’s sacrifice, the ileum was taken out and cleaned, one end was secured with a surgical thread, 100 μl of GEM-SAM-FVpE and FVpE were injected from the other end, and the tissue was securely tied. After 6 h of reaction, the tissue was fixed and then sectioned using a cryostat. The GEM-SAM-FVpE and FVpE were then identified using the Alexa Fluor^®^647-anti His antibody, while the PPs M cells were identified using the FITC-GP2 antibody. Examine the fluorescence signal using a confocal microscope (ZEISS, Germany).

### 2.5 Ability of GEM-SAM-FVpE vaccine to activate BMDCs *in vitro*

Bone marrow mononuclear cells were isolated from the femur and tibia of BALB/c mice (8 weeks) and cultured in RPMI 1640 medium containing 5% FBS, 1% penicillin-streptomycin, 20 ng/mL GM-CSF, 20 ng/mL IL-4 for 7 days to obtain BMDCs. To assess the activation capacity of the GEM-SAM-FVpE on BMDCs, cells were co-incubated with GEM, SAM-FVpE and GEM-SAM-FVpE for 24 h, respectively, and LPS were the positive control. Finally, the cells were collected and detected the expression of the surface markers or costimulatory molecules CD11c, MHC II, CD40, CD80, and CD86 by flow cytometry. Meanwhile, the supernatants of BMDCs co-incubated with the GEM-SAM-FVpE were collected and the secretion levels of IL-1β, IL-12p70, IL-4 and IL-6 were detected by ELISA kit.

### 2.6 Establishment of oral immunotherapy and therapeutic models

To evaluate the impact of GEM-SAN-FVpE on the immune response. 24 mice were divided into 4 groups, and were gavaged with PBS, GEM (1U), SAM-FVpE (100 μg), and GEM-SAM-FVpE (1U GEM with 100 μg antigen), respectively, once a week for 4 consecutive times. The serum, gastrointestinal lavage, feces and spleen were collected at 8 weeks for subsequent studies of immune mechanisms. About therapeutic models, 6-weeks SPF BALB/c male mice were randomly divided into 4 groups of 6 mice each. 3 groups were gavaged with 300 μL *H. pylori* (1 × 10^9^ CFUs/mL) every other day for 4 times, and the other group was given normal drinking water as a control. One month later, the *H. pylori*-infected mouse model was validated. Then, the 3 groups of infected mice were gavaged with PBS, antibiotic triple drug (50 mg/kg metronidazole, 25 mg/kg amoxicillin, 20 mg/kg omeprazole), and GEM-SAM-FVpE (1U GEM with 100 μg antigen) once a week for 4 consecutive weeks. 3 weeks after the last vaccination, stomach tissue and spleen were collected to analyze *H. pylori* clearance and splenic lymphocyte proliferative response.

### 2.7 Specific T lymphocytes and secreted cytokines are measured

Mouse splenic lymphocytes were isolated, after secondary stimulation with FVpE, detected by flow cytometry and ELISPOT. Meanwhile, the splenic cell supernatants were collected to detect the secretion levels of IFN-γ, IL-4, and IL-17A by ELISA.

### 2.8 Determination of specific antibodies produced in mice after oral immunization

FVpE antigen was coated with an ELISA plate at 2 μg/mL, blocked with 5% BSA-PBS the next day, and samples such as serum, gastrointestinal lavage and feces were added to the ELISA plate and incubated, followed incubated with mouse HRP-IgG, IgG1, IgG2a and sIgA. Finally, the color is developed and the reaction is terminated, reading the OD_450_.

### 2.9 Gastric mucosal sIgA inhibited *H. pylori* adhesion to GES-1 cells *in vitro*

GES-1 cells (2 × 10^5^ cells/well) were inoculated in 24-well plates and incubated at 37°C, 5% CO_2_ for 24 h. When the cells grew to 70%, *H. pylori* bacterial solution was added at MOI = 50, and PBS, SAM-FVpE, and GEM-SAM-FVpE gastric mucosal sIgA were added, respectively, and incubated for 12 h. After washing, *H. pylori* were fixed with methanol for 5 min, stained with Giemsa, and washed with water to observe *H. pylori* adherence under a microscope and count the number of *H. pylori* adhering on each cell. Meanwhile, total RNA was extracted by Trizol method, reverse transcribed into cDNA, and the expression of *H. pylori 16S rRNA* was detected by RT-qPCR, and primer sequences are shown in **Supplementary Table S1**.

### 2.10 Detection of *H. pylori* colonization in gastric tissue

3 weeks after the final treatment, stomach tissues were isolated and *H. pylori 16S rRNA* expression was detected by RT-qPCR. At the same time, the gastric tissues were fixed, dehydrated, embedded, sectioned, and immunohistochemically detected with anti-*H. pylori* antibody (Abcam, US) to observe under the microscope. In addition, the tissue sections were stained with hematoxylin-eosin to observe the damage and repair of gastric mucosal tissue, and Histological scoring criteria are shown in **Supplementary Table S2**.

### 2.11 Splenic lymphocyte proliferative response

Single-cell suspensions were prepared 3 weeks after the final treatment by isolating mouse spleens, adjusting the cell concentration to 5 × 10^6^ cells/mL, adding to 96-well plates (100 μL/well), and stimulating with fusion proteins (20 μg/mL), with no stimulant added to the negative control. incubation was performed for 72 h at 37 °C with 5% CO_2_, and 12 h prior to the end of the period, 3H-TdR (1 µL Ci/well) was added, and the incubation continued for 12 h. Cells were collected on filter paper using a cell collector, dried, and the value of pulses per minute (cpm) was determined by a liquid flash counter, and the results were expressed as the stimulation index (SI) (SI=mean value of cpm in experimental group/mean value of cpm in negative control group).

### 2.12 Statistical analysis

Statistical analysis was performed using GraphPad Prism 8.0 software, and results are presented as mean ± SD. Statistical significance was tested using t-test or two-way ANOVA test. *p < 0.05, **p < 0.01, ***p < 0.001, ns, *p*>0.05.

## 3 Results

### 3.1 Expression of recombinant proteins and preparation of GEM-SAM-FVpE

**Figure 1:**
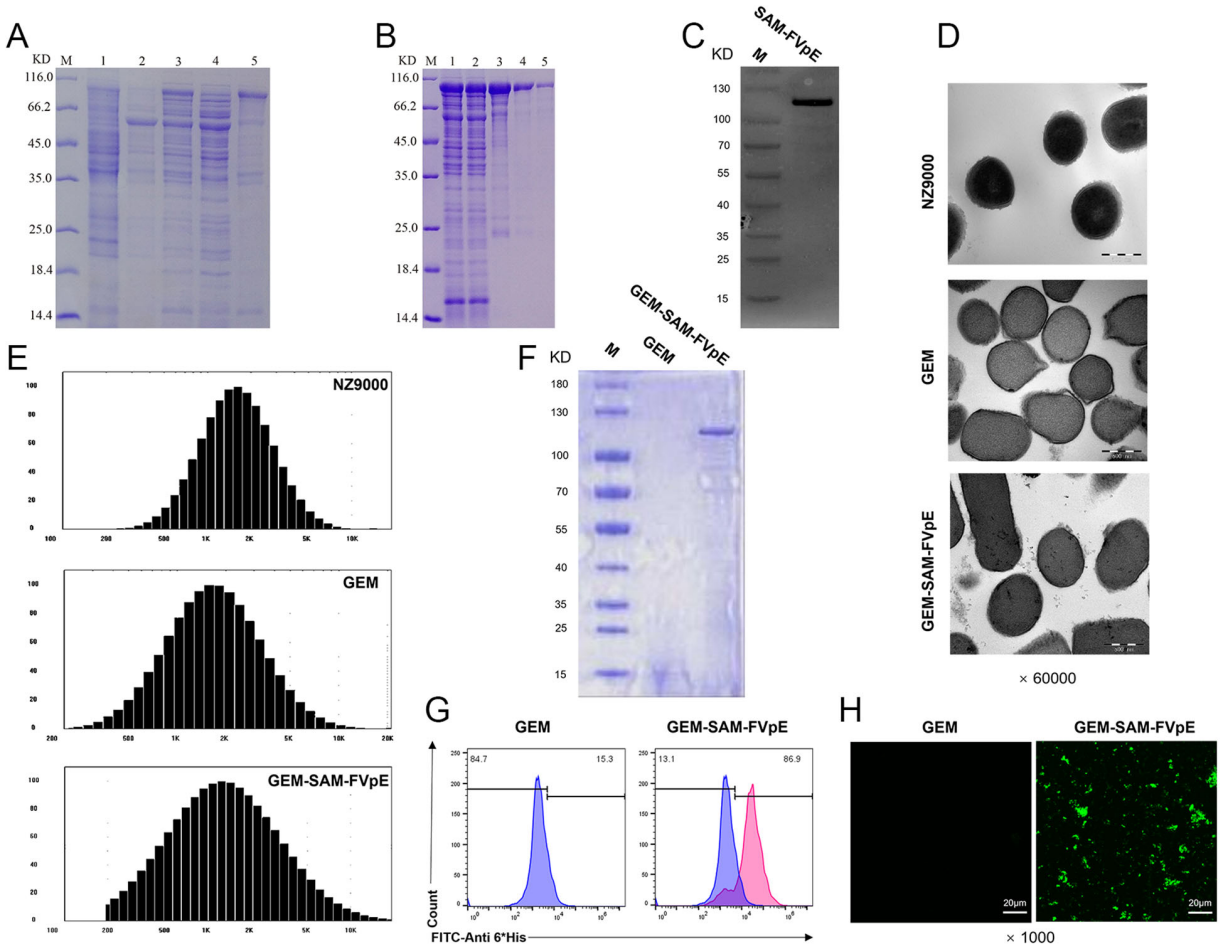
Expression of recombinant protein SAM-FVpE and preparation and identification of GEM-SAM-FVpE vaccine. **(A)** The expression of recombinant protein induced by 0.2 mM IPTG was analyzed by SDS-PAGE. (M: Protein marker; 1: The empty plasmid pCZN1 was induced by IPTG;2 and 3: *E. coli* BL21 (DE3) before and after induction with IPTG, respectively; 4: Lysate supernatant of induced *E. coli* BL21 (DE3); 5: Lysate precipitate of induced *E*. *coli* BL21 (DE3).). **(B)** SDS-PAGE analysis of Ni column purification of recombinant protein (M: Protein marker; 1: Sample after crushing; 2: Ni column outflow sample; 3-5: Samples after multiple elution.). **(C)** Western blot identification of recombinant protein. **(D)** Morphology of NZ9000, GEM and GEM-SAM-FVpE under the TEM (×60000, Scale bar: 500 nm). **(E)** Particle size of NZ9000, GEM and GEM-SAM-FVpE. **(F)** SDS-PAGE analysis of SAM-FVpE recombinant protein on the surface of GEM. **(G)** The efficiency of recombinant protein SAM-FVpE binding on surface of GEM by flow cytometry. **(H)** Immunofluorescence analysis of SAM-FVpE recombinant protein on the surface of GEM (×1000, Scale bar: 20 µm).

We inserted the gene sequence of FVpE into the plasmid vector to obtain the recombinant plasmid pCzn1-SAM-FVpE, and the recombinant antigen SAM-FVpE was obtained after prokaryotic induced expression (**Figure 1A**), which was subsequently purified by affinity chromatography on Ni-NTA columns. The SDS-PAGE and western blot results confirmed the success in obtaining the purified target protein SAM-FVpE (**Figures 1B, C**). To verify whether the GEM particles were successfully prepared, TEM was used to study the morphological differences between live lactic acid bacteria NZ9000 and GEM particles. It was found that untreated NZ9000 had a more uniform internal structure and the cytoplasm was darker in color. While the GEM particles had less content, but still maintained an intact peptidoglycan structure (**Figure 1D**). Moreover, the size of GEM particles did not changes significantly compared with NZ9000, and both remained between 1000–2500 nm (**Figure 1E**). Subsequently, after confirming the optimal binding ratio between GEM and SAM-FVpE antigen (100 μg SAM-FVpE per 1 U GEM) (Supporting Information, **Supplementary Figures S1**, **S2**), we prepared the GEM-SAM-FVpE vaccine. The TEM revealed evenly distributed filamentous substances, indicating successful binding of SAM-FVpE to GEM particles (**Figure 1D**). SDS-PAGE, Flow cytometry and immunofluorescence analysis estimated that SAM-FVpE successfully displayed on the surface of GEM, with a display efficiency of 90% (**Figures 1F–H**).

### 3.2 Validation M cell-targeting of GEM-SAM-FVpE

**Figure 2:**
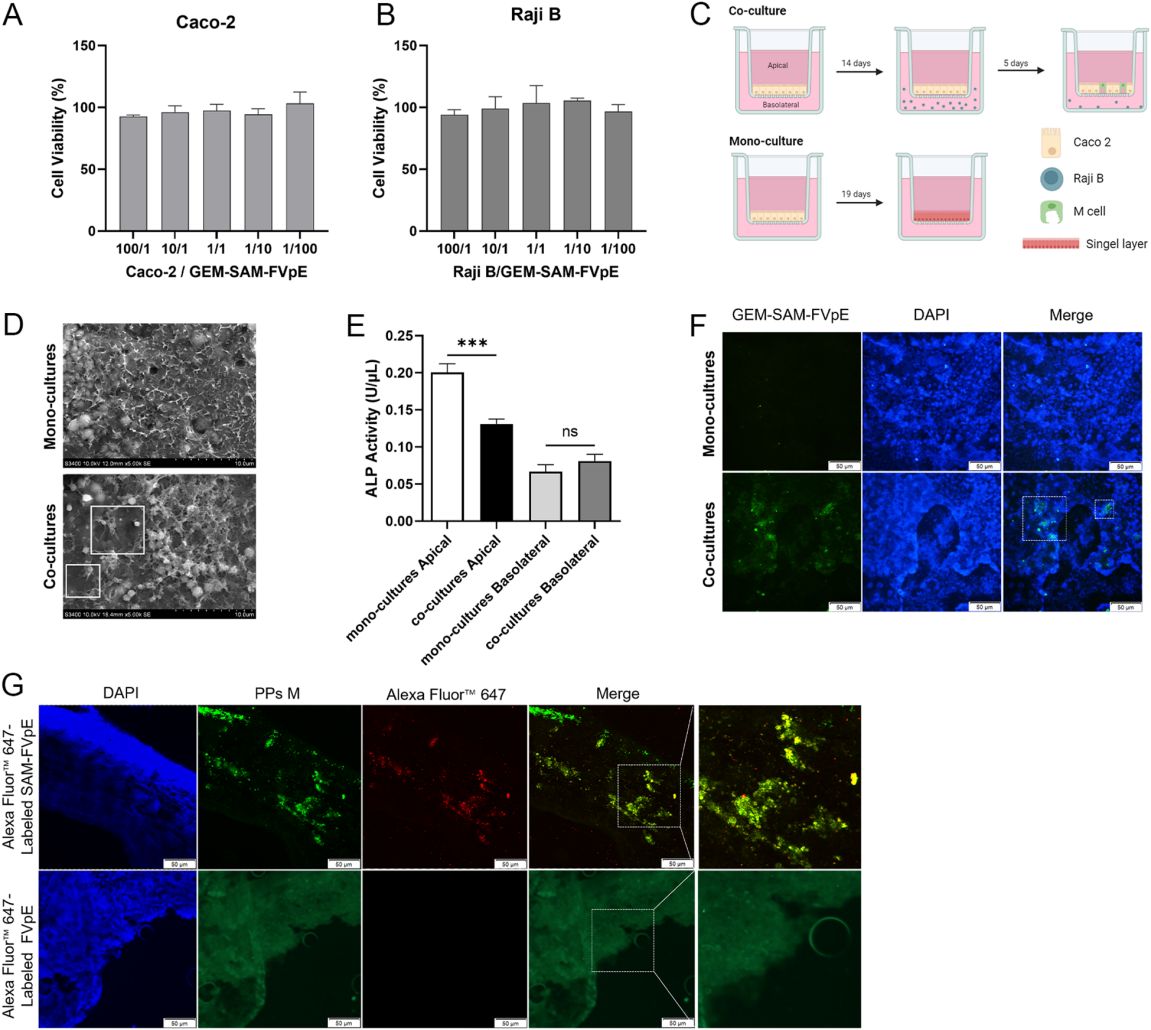
Validation of M-cell targeting of GEM-SAM-FVpE vaccine. **(A, B)** Cell viability of GEM-SAM-FVpE vaccine on Caco-2 and Raji B cells. **(C)** Schematic diagram of building an *in vitro* model of M cells. **(D)** Morphology of M cells model under the scanning electron microscopy (×5000, Scale bar: 10 µm). **(E)** Changes of alkaline phosphatase (ALP) activity on Apical and Basolateral sides of M cells model. **(F)** The targeting of GEM-SAM-FVpE vaccine was verified by M cells model (×200, Scale bar: 50 µm). **(G)** The targeting of GEM-SAM-FVpE vaccine was verified by ileal closed-loop experiment (×200, Scale bar: 50 µm). *p < 0.05, **p < 0.01, ***p < 0.001, ns, p>0.05.

Multiple studies have shown that the fusion of M cell targeting peptides with the target antigen can be specifically recognized by M cells, improve the uptake efficiency of oral vaccines, thereby evoking antigen-specific systemic and mucosal immune responses (28–30). Firstly, the GEM-SAM-FVpE vaccine had no significant effect on the cell viability of Caco-2 and Raji B cells (**Figures 2A, B**). In order to verify the M cell-targeting properties of GEM-SAM-FVpE vaccine, an M cell model was constructed using co-culture of Caco-2 cells and Raji B cells (**Figure 2C**). Observation of cell morphology in SME revealed short and irregular microvilli on the cell surface in the co-culture model (**Figure 2D**). And in co-culture, the ALP activity in the apical decreased significantly compared with mono-cultures, which was related to the formation of microvilli, indicating that Caco-2 cells co-cultured with Raji B cells form M-cells-like morphological characteristics (**Figure 2E**). Next, we labeled the GEM-SAM-FVpE vaccine with AlexFluor^®^ 488-Anti His antibody, and the immunofluorescence results showed significant fluorescent signals in the co-culture with the same number of particles, demonstrating that the GEM-SAM-FVpE *in-vitro* was able to target the M cell model (**Figure 2F**). Finally, mice ileal loop assay showed that GEM could assist the SAM-FVpE fusion protein to target M cells under the action of Mtp (**Figure 2G**).

### 3.3 Activation and maturation of BMDCs induced by GEM-SAM-FVpE

**Figure 3:**
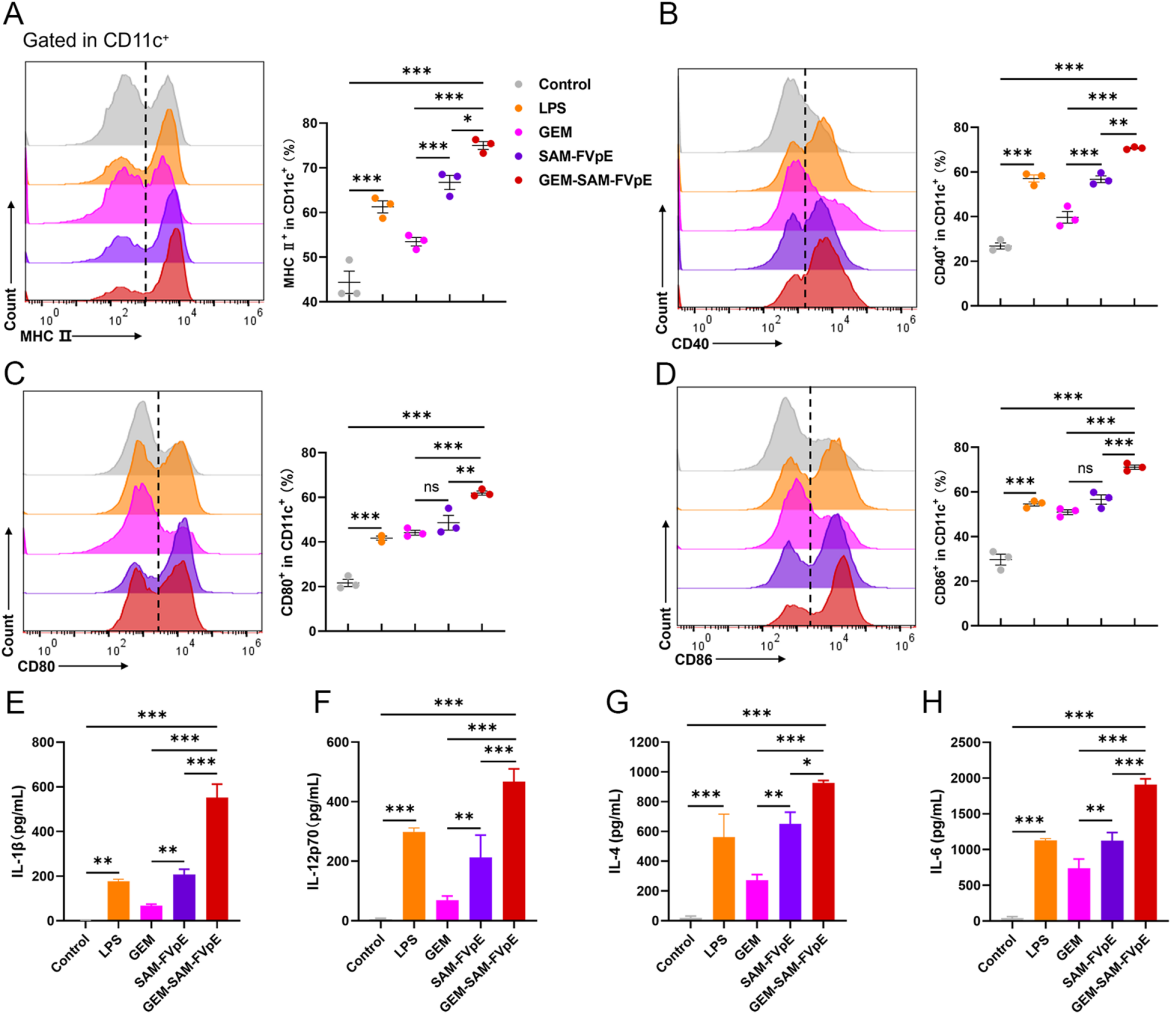
GEM-SAM-FVpE vaccine induces sustained activation and maturation of BMDCs. **(A)** MHC II, **(B)** CD40, **(C)** CD80 and **(D)** CD86 expressions in CD11c^+^ BMDCs induced by GEM-SAM-FVpE vaccine (n=3). BMDCs were exposed to GEM-SAM-FVpE vaccine for 24 **(h)** LPS, GEM, and recombinant protein SAM-FVpE were used as controls. **(E)** IL-1β, **(F)** IL-12, **(G)** IL-4 and **(H)** IL-6 secretions induced by GEM-SAM-FVpE vaccine (n=3). *p < 0.05, **p < 0.01, ***p < 0.001, ns, p>0.05.

To evaluate the immune efficacy of the vaccine, we studied the ability of the GEM-SAM-FVpE to activate mice bone marrow-derived dendritic cells (BMDCs), a key step in initiating the adaptive immune response. BMDCs were co-incubated with GEM, SAM-FVpE and GEM-SAM-FVpE for 24 h, respectively, and LPS-treated BMDCs were used as control. The maturation of BMDCs was evaluated using flow cytometry to detect the expression of cell surface markers or costimulatory molecules in each group. The results showed that compared with the Control group, SAM-FVpE and GEM-SAM-FVpE were able to significantly increase the expression of the co-stimulatory molecules MHC II, CD40, CD80, and CD86. In addition, GEM-SAM-FVpE was more capable of activating DCs compared to SAM-FVpE (**Figures 3A–D**). Furthermore, mature DCs have enhanced antigen presentation ability to activate T cell function. To evaluate the ability of DCs to secrete cytokines after different treatments, we used ELISA to measure the IL-1β, IL-12p70, IL-4, and IL-6 in the cell supernatant. The results are shown in **Figures 3E–H**, GEM-SAM-FVpE enhanced the stimulation of DCs to secrete cytokines IL-1β, IL-12p70, IL-4, and IL-6, and increased the ability of the vaccine to induce specific CD4^+^ T lymphocyte responses.

### 3.4 Robust humoral and mucosal immune responses induced by GEM-SAM-FVpE

**Figure 4:**
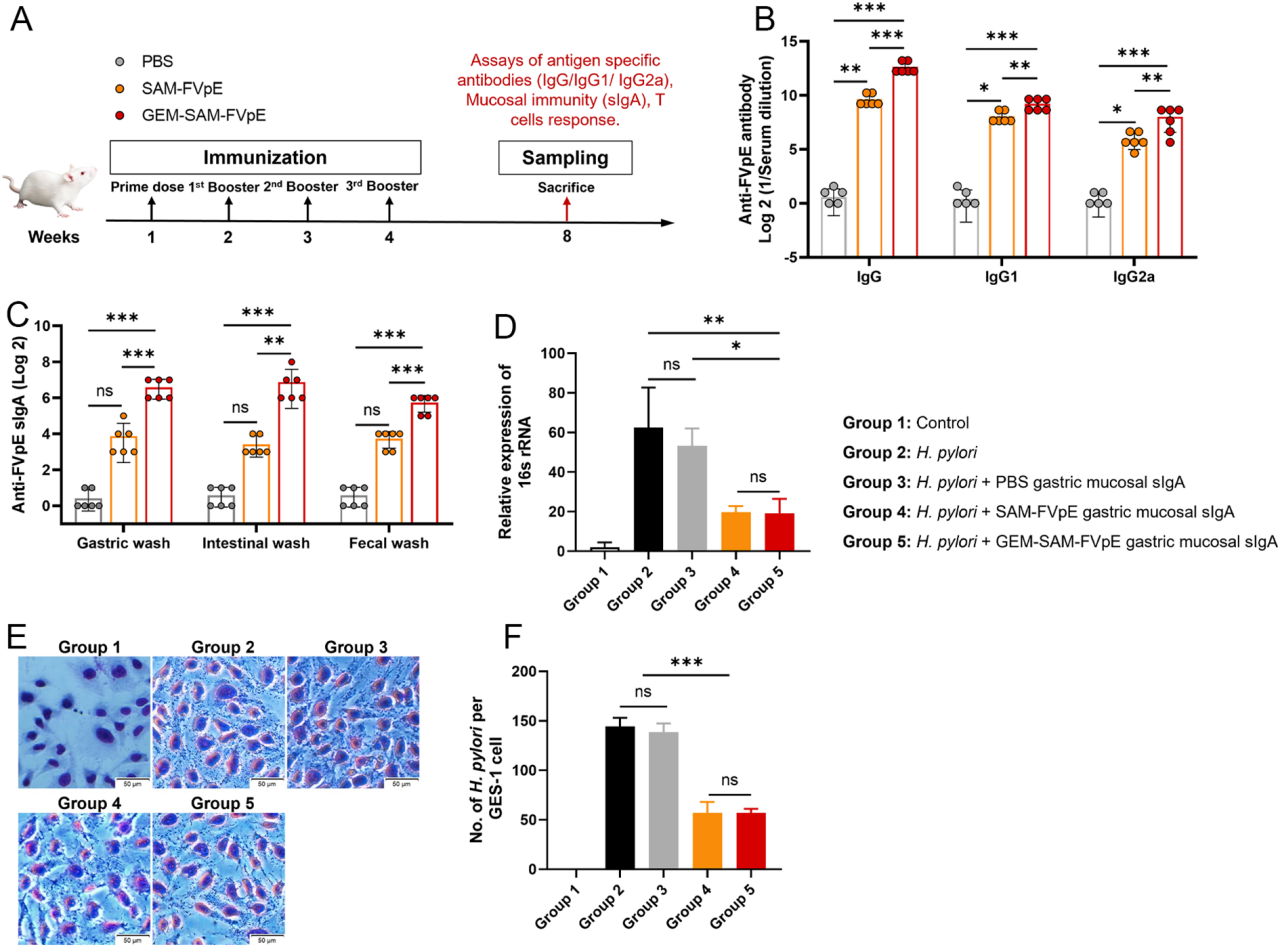
GEM-SAM-FVpE vaccine induced a robust humoral response after oral immunization. **(A)** Schematic illustration of the experimental design. BALB/c mice were immunized with 10 µg recombinant protein SAM-FVpE and GEM-SAM-FVpE vaccine formulated with 10 µg SAM-FVpE protein, PBS were used as control every other week for four consecutive immunizations (n=6). **(B)** Serum samples were collected on weeks 8, and the total amount of anti-FVpE IgG was quantified with ELISA. **(C)** Gastric wash, intestinal wash and fecal wash samples were collected on weeks 8, and the total amount of anti-FVpE sIgA was quantified with ELISA. **(D-F)** Gastric wash samples neutralize/inhibit the adhesion of *H*. *pylori* to normal gastric epithelial GES-1 cells *in vitro* (n=3). After gastric mucosal sIgA, *H. pylori* and GES-1 were co-cultured for 12 h, **(D)** the expression of *H*. *pylori 16S rRNA* was detected by RT-qPCR, and **(E, F)** the adhesion of *H*. *pylori* numbers on GES-1 cells was observed by Giemsa staining (×200, Scale bar: 50 µm). *p < 0.05, **p < 0.01, ***p < 0.001, ns, p>0.05.

To evaluate the adaptive immunogenicity of GEM-SAM-FVpE, mice were orally administered with PBS, single antigen SAM-FVpE and GEM-SAM-FVpE respectively, and immunized every other week for 4 consecutive times, and samples were collected at week 8 (**Figure 4A**). ELISA was performed to detect FVpE-specific IgG and its subtypes (IgG1, IgG2a) in serum, and the levels of IgG, IgG1, and IgG2a antibodies in the single-antigen SAM-FVpE group and GEM-SAM-FVpE group were higher than those in the PBS group, and the increasing effect was more obvious in the GEM-SAM-FVpE group (**Figure 4B**). *H. pylori* cause chronic inflammation and proliferation of abnormal epithelial cells by colonizing the gastric mucosa, which in turn increases the risk of gastric ulcers and gastric cancer (31, 32). Therefore, it is crucial to evaluate the mucosal immunization effect induced by oral vaccines, that is, the level of sIgA antibody production. We measured the antigen-specific sIgA produced in gastric mucosa, intestinal mucosa and feces respectively, the production of sIgA in the GEM-SAM-FVpE group was significantly higher, and the effect was more significant than that in the single-antigen SAM-FVpE group, but there was no significant difference in the single-antigen SAM-FVpE group compared with the Control group (**Figure 4C**). The above results shown that compared with single antigen SAM-FVpE, under the protective effect of the carrier, GEM-SAM-FVpE was able to deliver the antigen to the immune effector site and elicite stronger mucosal and systemic immune responses. Next, to test the effect of mouse gastric mucosal sIgA antibody on inhibiting the adhesion of *H. pylori* to normal gastric epithelial cells GES-1 *in vitro*, the collected PBS, SAM-FVpE, and GEM-SAM-FVpE murine gastric mucosal sIgA antibodies were added simultaneously to the *H. pylori* + GES-1 co-culture model, respectively, analyzed by RT-qPCR and Giemsa staining after 12 h incubation. RT-qPCR to detect the expression of *H. pylori* specific *16S rRNA* in the samples and Giemsa staining to direct count the numbers of *H. pylori* adhering to each GES-1. The results demonstrated that both single-antigen SAM-FVpE and GEM-SAM-FVpE groups of gastric mucosal sIgA significantly reduced *H. pylori* adherence to GES-1 *in vitro* compared with the control group (**Figures 4D–F**). Although there was no significant difference between the single-antigen SAM-FVpE group and the GEM-SAM-FVpE group, this may be because the gastric mucosal sIgA produced by the two groups inhibited *H. pylori* adherence, and there was no association with the titers of the antibodies.

### 3.5 Robust CD4^+^ T cell responses induced by GEM-SAM-FVpE

**Figure 5:**
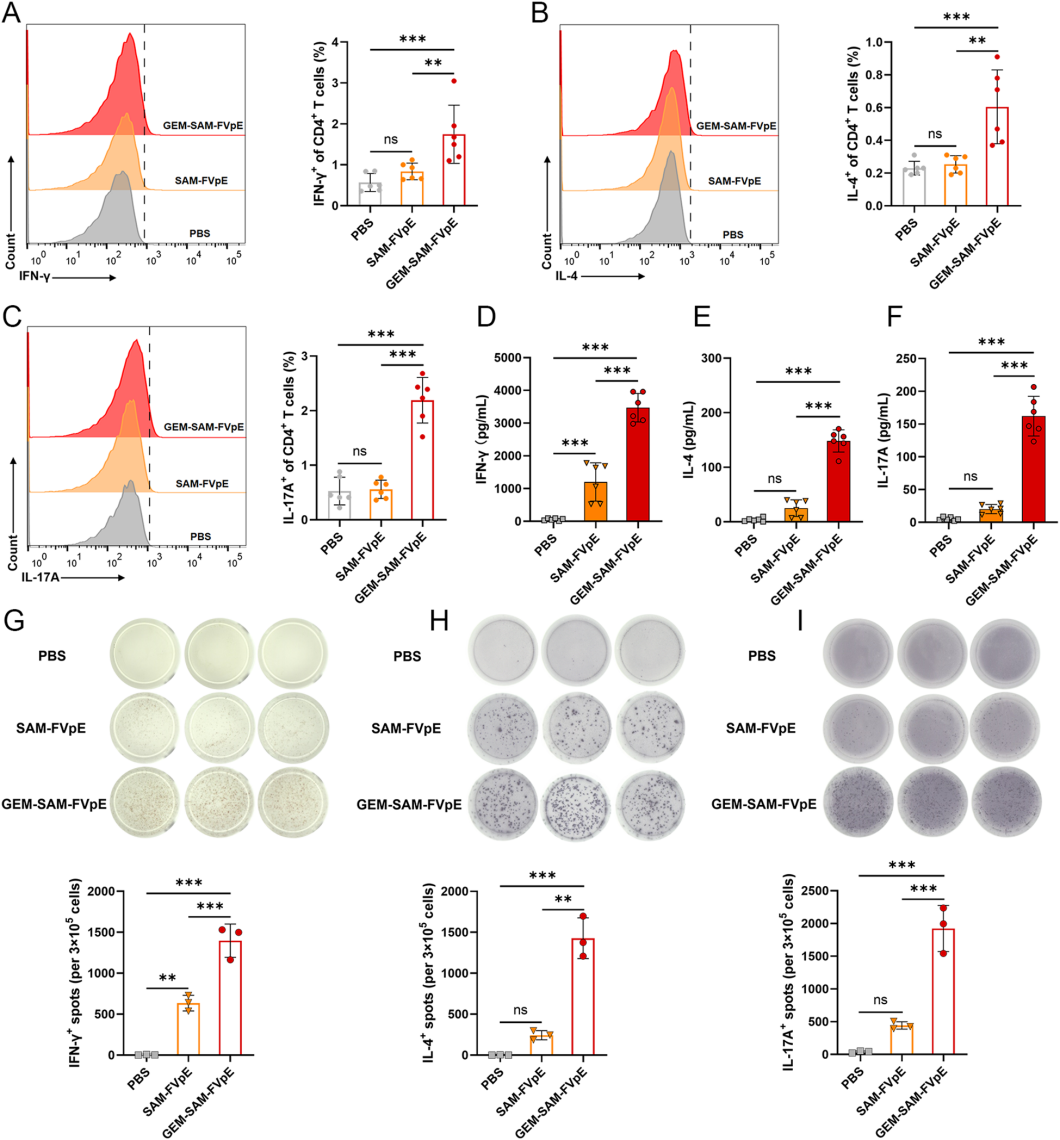
GEM-SAM-FVpE vaccine elicited robust specific CD4^+^ T-cell responses. Splenic lymphocytes were collected on weeks 8, and the FVpE specific CD4^+^ T cells was quantified with flow cytometry, ELISA and ELIspot. **(A–C)** Changes in the proportion of IL-4, IFN-γ and IL-17-screting CD4^+^ T cells by flow cytometry. **(D–F)** The levels of IFN-γ, IL-4, and IL-17 in the supernatant of splenic lymphocytes after FVpE antigen restimulation were measured by ELISA. **(G–I)** The number of IFN-γ, IL-4, and IL-17 spot-forming cells in spleen lymphocytes restimulated 72 h with FVpE antigen by ELISpot assay (n=3). *p < 0.05, **p < 0.01, ***p < 0.001, ns, p>0.05.

Since vaccine-mediated T-cell responses are essential for preventing and curing *H. pylori* infections, we were the first to confirm that GEM-SAM-FVpE can start antigen presentation by activating dendritic cells. We conducted the following tests to find out if the vaccines given orally may trigger antigen-specific Th cell responses: mice were gavaged PBS, SAM-FVpE, and GEM-SAM-FVpE once a week for four weeks, respectively. After isolating mouse splenic cells at week 8 and subsequently stimulating them with antigen *in vitro*, and IFN-γ, IL-4, and IL-17A were selected as detection indicators, corresponding to Th1, Th2, and Th17 reactivity, respectively, flow cytometry, ELISA, and ELISPOT were used to evaluate the reactivity of GEM-SAM-FVpE to CD4^+^ T cells (**Figure 4A**). Flow cytometry results showed that GEM-SAM-FVpE could significantly increase the percentages of Th1, Th2 and Th17 lymphocytes, while the single antigen SAM-FVpE group only induced Th1 responses (**Figures 5A–C**). In addition, to fully test our hypotheses with multidimensional experimental data, ELISA detected the cytokines IFN-γ, IL-4, and IL-17A secreted by splenic lymphocytes, and the results were consistent with the flow cytometry results (**Figures 5D–F**). Similarly, ELISPOT showed that the number of IFN-γ, IL-4, and IL-17 spot-forming cells was significantly increased in the GEM-SAM-FVpE group (**Figures 5G–I**). The above results indicated that GEM-SAM-FVpE induced a more favorable helper T-cell response.

### 3.6 Therapeutic efficacy of GEM-SAM-FVpE vaccine

**Figure 6:**
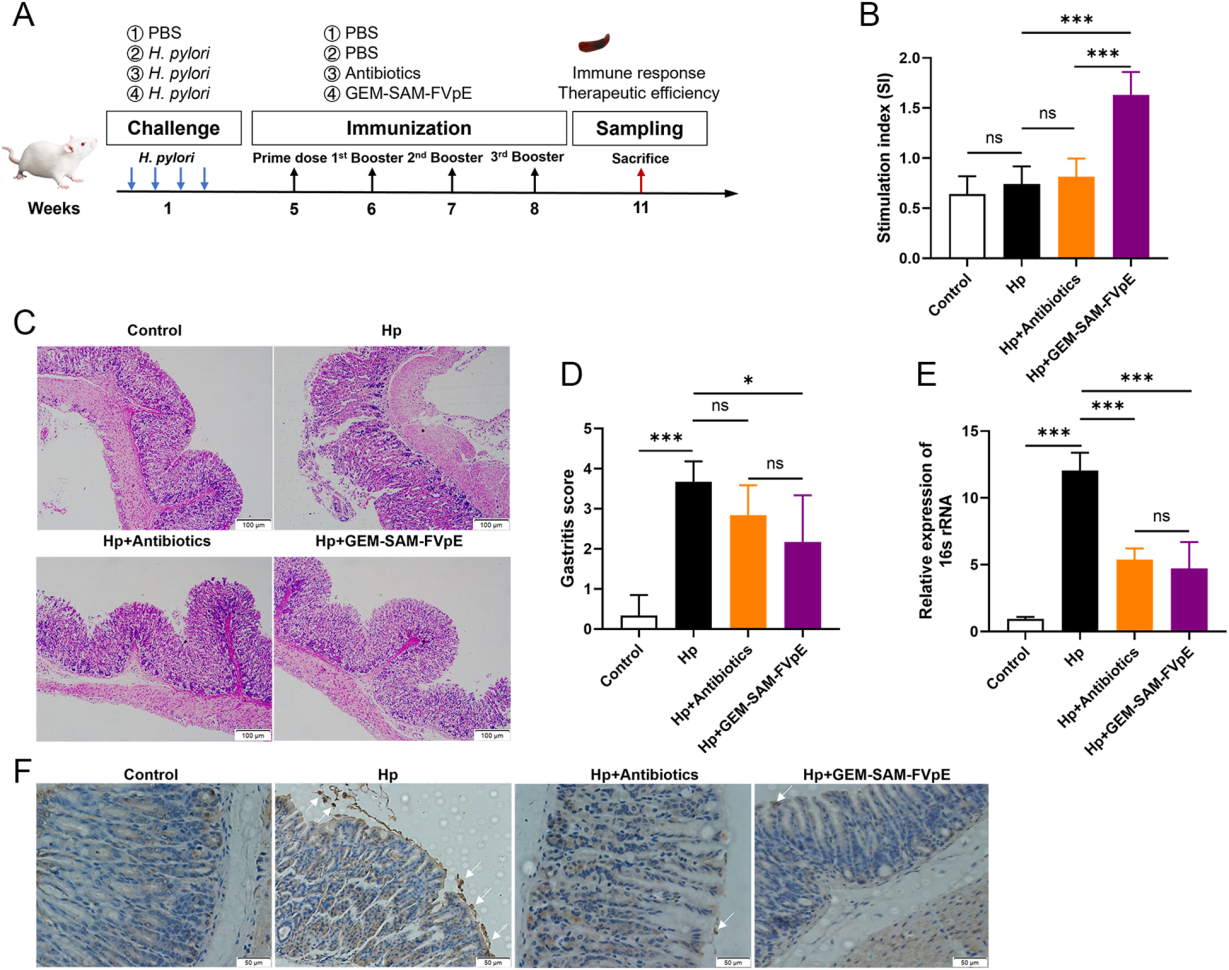
GEM-SAM-FVpE vaccine effectively eliminated (*H*) *pylori* colonization in gastric tissue. **(A)** Schematic diagram of the therapeutic vaccination. BALB/c mice were infected with *H*. *pylori* SS1. One month later, infected mice were immunized with GEM-SAM-FVpE vaccine. Mice immunized with PBS or Antibiotics (amoxicillin, 50 mg/kg; clarithromycin, 25 mg/kg; omeprazole, 20 mg/kg) were used as controls. **(B)** Proliferative response of T lymphocytes. After stimulation with *H*. *pylori* lysates, the proliferation of splenic T lymphocytes was examined. **(C, D)** Histological evaluation of gastric tissues using H&E staining (×100). **(E)** *16S rRNA* expression levels in gastric mucosal tissues were examined via RT-qPCR. As an internal control, 18S rRNA was used for the expression of *H*. *pylori 16S rRNA*. **(F)** Immunohistochemical analysis of *H*. *pylori* in gastric mucosa (×200). *p < 0.05, **p < 0.01, ***p < 0.001, ns, p>0.05.

We established an *H. pylori* mouse infection model according to **Figure 6A** to evaluate the therapeutic effect of GEM-SAM-FVpE. Subsequently, PBS, antibiotic triple drug (50 mg/kg metronidazole, 25 mg/kg amoxicillin, 20 mg/kg omeprazole) and GEM-SAM-FVpE were administered orally. 2 weeks after the last treatment, mouse spleen lymphocytes were extracted and stimulated with *H. pylori* lysate to compare the effects of different treatments on the proliferative response of T lymphocytes and expressed as stimulation index (SI). As shown in **Figure 6B**, the lymphocyte proliferation levels in the GEM-SAM-FVpE group were significantly higher than those in other groups. In addition, the gastric mucosal damage in mice with different treatments was evaluated by HE staining. The findings demonstrated that there was substantial pathological damage to the stomach tissues of *H. pylori*-infected mice, as demonstrated by the mucosal layer becoming significantly thinner, the glandular structure being nearly entirely lost, and the infiltration of numerous inflammatory cells. But mice given GEM-SAM-FVpE had a notable decrease in gastric mucosal damage, as demonstrated by a notable decrease in inflammatory cell infiltration, a partial recovery of the mucosal layer thickness, and a degree of glandular structural repair (**Figures 6C, D**). Finally, in order to evaluate the efficiency of GEM-SAM-FVpE in clearing *H.pylori* from the gastric tissue of infected mice, RT-qPCR and immunohistochemistry were used to detect *H. pylori*-specific *16S rRNA* and *H. pylori* colonization in the gastric tissues. The results all showed that both GEM-SAM-FVpE and antibiotics could significantly reduce *H. pylori* in gastric tissue, but the therapeutic effect of GEM-SAM-FVpE was superior, which may be related to the fact that antibiotic treatment caused recurrent infections with repeated treatments. (**Figures 6E, F**).

## 4 Discussion

*H. pylori* is a microaerophilic Gram-negative bacterium that is able to colonize the acidic gastric mucosal environment because it secretes urease that converts urea into ammonia (33, 34). Long-term infection with *H. pylori* triggers chronic inflammation, gastric ulcers, gastric cancer and other gastric diseases, and is currently recognized as the most relevant risk factor for gastric cancer (35–37). Thus, the prevention and eradication of *H. pylori* have become a global challenge. Currently, the main clinical treatment method is combination therapy based on antibiotics (38). However, a series of problems such as the resistance of *H. pylori* caused by long-term use of antibiotics have also emerged (39). The mucosal immune barrier is the first line of defense of the immune system (40), and that’s why mucosal immunity induced by the use of *H. pylori* specific antigens via the oral route promises to be an important alternative or antibiotic supplemental therapy. The colonization and infection of *H. pylori* is largely dependent on its multiple virulence factors, so our team designed a multivalent epitope vaccine, FVpE, containing functional fragments: NAP, CagA, VacA, and a urease multi-epitope peptide (UE) from CTB-UE. However, due to the harsh acidic environment of the gastrointestinal tract, the recombinant vaccines suffer from poor antigenic stability and difficulty in eliciting a potent immune response. Suitable adjuvants and carriers are urgently needed to deliver it to the immune response site and exert stronger immune efficacy.

Selection of an appropriate vaccine delivery system is one of the core elements of vaccine development. GEM particles are non-living bacterial carriers based on the cell wall skeleton of *L. lactis*, which form a hollow granular matrix by removing their own contents and surface proteins. GEM particles have shown unique advantages in the vaccine delivery field because of their combination of biosafety and efficient immune activation (41, 42). *L. lactis* are particularly suitable for oral vaccines by virtue of their high resistance to gastric acid, digestive enzymes and temperature to avoid antigen degradation during delivery. As a result, we adopted a *L. lactis*-based GEM particle surface display system, which non-covalently binds the antigen SAM-FVpE fused with cA to the GEM surface by high affinity. TEM revealed that after thermal acid treatment removed macromolecules such as proteins and nucleic acids from their cells, and retained their intact peptidoglycan structure and cytoskeleton. Compared to live bacterial vaccines, GEM particles overcome safety concerns caused by genetic modification while retaining the advantages of *L. lactis* carriers. Moreover, the delivery system has a higher antigenic load. The GEM surface display system has addressed the key problems such as poor targeting and single immune activation of traditional vaccine carriers. Additionally, GEM was applied to the treatment or prevention of different diseases by loading antigens of pathogens. In recent years, GEM has demonstrated great potential in infectious diseases such as sudan virus (SUDV) (43), human papillomavirus (HPV) (44), and Middle East Respiratory Syndrome-associated coronavirus (45), and is progressively making inroads into cancer immunotherapy.

**Scheme 2:**
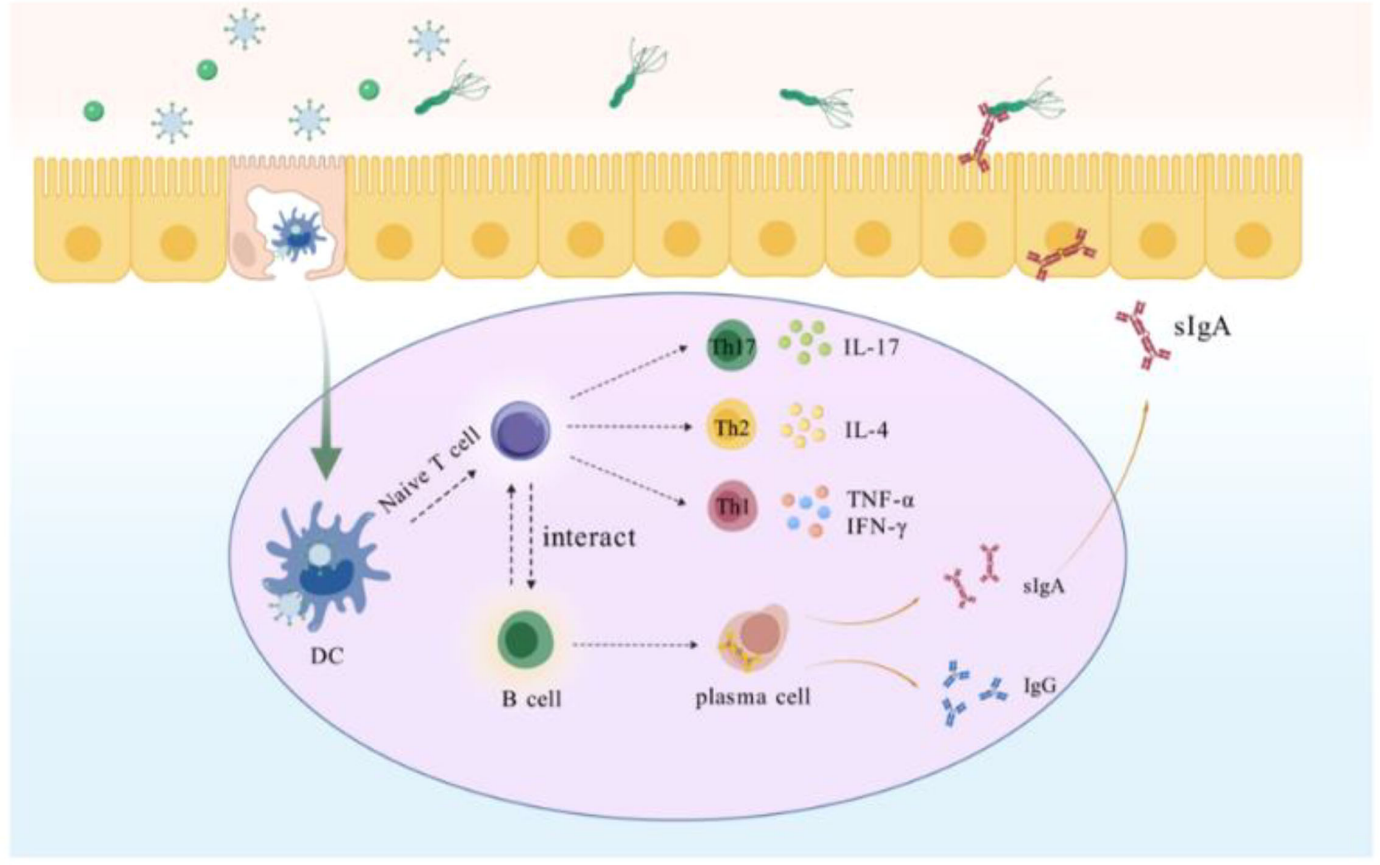
Within the gut-associated follicular epithelium M cells directly recognize GEM-SAM-FVpE and transport it to DCs, which are processed by DCs for vaccine to activate T cells, which further differentiate into Th cells. Th1 secretes IFN-γ and TNF-α, which assists cellular immunity to promote killing *H. pylori*; Th2 secretes IL-4, which assists humoral immunity; and Th17 against *H. pylori* by stimulating multiple cells to participate in the collective immune defense through the secretion of IL-17 and others. In addition, GEM-SAM-FVpE stimulates B cells located in the lamina propria of the mucosa to differentiate into plasma cells, promotes antibody switching, and induces the production of sIgA and IgG. sIgA is secreted into the gastric mucosa site and inhibits *H. pylori* adherence through the neutralization of virulence factors; IgG enters the bloodstream to resist *H. pylori* infection and can persist for months or years.

M-cell targeting strategies are a key component in enhancing mucosal immunity and resistance to pathogens. M cells located in Peyer’s patches can effectively bind and deliver biological macromolecules, including microbial antigens (46, 47). These antigens endocytosed by M cells are presented to APCs, which process and present them to T and B lymphocytes to elicit an adaptive immune response (48, 49). This process not only shortens the time for antigen presentation, but also improves the utilization of the antigen. Hence, designing M-cell-targeting antigens can enhance the antigen uptake by M-cells, thereby inducing a high level of mucosal immune response, that will be a more effective and attractive new strategy for eliminating *H. pylori* (**Scheme 2**). In this study, we designed GEM-SAM-FVpE to target M cells via Mtp to induce effective mucosal immunity and adaptive immune responses for the treatment of *H. pylori* infection. So far, the *in vitro* M cells model constructed using the co-culture of Caco-2 cells and Raji B cells have been widely used to explore oral drug permeability, vaccine transport mechanisms (50, 51). After constructing the M cells model, we confirmed that GEM-SAM-FVpE can target M cells model. Not only that, combined with the ileal loop assay also confirmed the M cells targeting ability of GEM-SAM-FVpE *in vivo* perspective. After GEM-SAM-FVpE is transported by M cells, whether APCs can be effectively activated is the next issue we need to discuss.

Dendritic cells (DCs) are the most important APCs, which regulating the immune response by ingesting, processing and presenting antigens to T cells, while expressing costimulatory molecules and cytokines (52, 53). Multiple studies have shown that GEM can activate and promote the maturation of DCs both *in vivo* and *in vitro*, allowing them to exhibit increased capacity to secrete pro-inflammatory cytokines, Th1-type cell-polarizing cytokines, antigen presentation, and stimulation of CD4^+^/CD8^+^ T cells (12). Moreover, GEM can also induce the expression of the chemokines CCL-20 and TSLP in DCs. Thus, could loading antigens onto the surface of GEM enhance the function of DCs? Therefore, after co-culturing GEM-SAM-FVpE with BMDCs, we detected the expression of MHC II and co-stimulatory molecules CD80, CD86, and CD40. It was found that compared to the SAM-FVpE, GEM-SAM-FVpE was able to significantly upregulate the expression of these markers, demonstrating a better ability to promote DCs maturation. At the same time, the effect of GEM-SAM-FVpE on Th cell was evaluated by detecting the secretion of cytokines IL-1β, IL-12p70, IL-4, and IL-6 cytokines. The results showed that the secretion of cytokine was stimulated to be significantly higher in the GEM-SAM-FVpE group than that in the SAM-FVpE group. Consequently, based on the above research results, we confirmed that GEM-SAM-FVpE efficiently delivers the vaccine to APCs by targeting M cells to initiate mucosal immunity and adaptive immune responses.

lgG are the main serologic marker of *H. pylori* infection, appearing within 23 weeks of infection and persisting for months to years, and are widely used for epidemiologic screening and clinical diagnosis. IgG subclasses reflect the Th1/Th2 immune polarization state and are correlate with infection outcomes and immune protection (54). After GEM-SAM-FVpE immunizing, we detected antigen-specific IgG and their subtypes (IgG1, IgG2a) in the serum of the mice. We found that GEM-SAM-FVpE was able to significantly increase the antibody titers, suggesting that the GEM can effectively induce the generation of high levels of humoral immune response. Furthermore, the key to oral immunization is producing high levels of sIgA to induce mucosal immune responses, which hinder the colonization of *H. pylori*. In this study, by measuring the specific sIgA titers in gastric lavage, intestinal lavage, and feces, it was found that GEM-SAM-FVpE can significantly stimulate mucosal immune responses. Meanwhile, the gastric mucosal sIgA can inhibit the adhesion of *H. pylori* to normal gastric epithelial cells GES-1 *in vitro*. Specifically, the mucosal sIgA generated by GEM-SAM-FVpE immunity can bind to the virulence proteins of *H. pylori* that mediate adhesion, exerting a neutralizing effect and inhibiting the adhesion of *H. pylori*. CD4^+^ T cells plays an important role in the immune response, regulation, and pathology process of *H. pylori* infection, and vaccines and immunotherapies targeting their response mechanisms have potential applications (55). Studies have shown that transferring splenic CD4^+^ T cells from immunized mice to immunodeficient mice can protect mice from *H. pylori* infection, confirming that T cell-mediated immune mechanisms play an important role (56, 57). Based on their cytokine production and function, CD4^+^ T cells can be categorized into Th1, Th2, Th17 and Treg. In this study, we used flow cytometry, ELISPOT, and ELISA to detect the CD4^+^ T cell response 8 weeks after immunization with GEM-SAM-FVpE. The results demonstrated that GEM-SAM-FVpE activated Th1, Th2, Th17 more effectively than mono antigen, playing a role in eliminating *H. pylori* and preventing inflammatory damage. Finally, the therapeutic effect of GEM-SAM-FVpE was evaluated by constructing a *H. pylori* infection model and gave appropriate GEM-SAM-FVpE treatment, detecting the repair of gastric tissue damage, the expression of *16S rRNA*, and the *H. pylori* colonization in gastric tissue. The results showed that GEM-SAM-FVpE mice exhibited a significant reduction in bacterial load and gastric mucosal damage after *H. pylori* infection, confirming the effectiveness and feasibility of this vaccine strategy.

In this study, our most critical innovation is the use of GEM delivery vehicle, combined with M cell targeting strategies, to achieve more efficient antigen delivery and immune activation. However, when we assess the safety and effectiveness of vaccines, analyze the potential challenges, there are still a number of factors that need to be taken into account to ensure that the results are scientifically and reliable. First, the host immune response to *H. pylori* infection shows a high degree of heterogeneity, and some populations may exhibit immune tolerance rather than protective immunity, resulting in variable vaccine efficacy in different populations. Second, inter-individual differences in M cell distribution and function may affect the immune effect of the vaccine. Therefore, the long-term protective effect of the vaccine and its effectiveness in different populations still need to be further validated. To address these challenges, we should further optimize the vaccine design, such as developing more specific M-cell-targeting ligands or combining multiple antigenic epitopes to improve the broad-spectrum and durability of the vaccine. In addition, the translation from medical research to clinical practice is even more challenging.

## 5 Conclusion

In summary, this study constructed the M cell-targeting vaccine GEM-SAM-FVpE, which loaded with *H. pylori* multi-epitope antigens as an oral vaccine. As far as its carrier is concerned, GEM is derived from *L. lactis* that meet food-grade standards, and it is a postbiotic-based preparation, unaffected by its own genetic material and potentially biocompatible. Another advantage of GEM is that it loads exogenous antigens on the surface, making it more stable than free antigens and less susceptible to degradation by stomach acid and various proteases. The *H. pylori* multi-epitope antigen SAM-FVpE was obtained by prokaryotic induction expression and purification, displayed on the surface of GEM under the cA with good immunogenicity high display efficiency. *In vivo* studies have confirmed that the GEM-SAM-FVpE can activate antigen-specific immune responses after oral immunization, and further eliminate the colonization of *H. pylori* at the gastric mucosal site by inducing obvious mucosal immunity (specific sIgA). Overall, GEM is a potential carrier that can deliver *H. pylori* antigens to M cells to initiate mucosal immune response, and it is easier to be generalized for application and has high safety. This will become a new approach for mucosal vaccine development with a broad promising clinical application. In addition, the design of GEM-SAM-FVpE provides new ideas for the prevention and control of *H. pylori* infection and is expected to play an important role in improving global public health.

## Data Availability

The datasets presented in this article are not readily available because The original contributions presented in the study are included in the article. Further inquiries can be directed to the corresponding author. Requests to access the datasets should be directed to guoletian1982@163.com.
