# Supplementary material for: Gram-positive enhancer matrix delivering FVpE via M cell targeting elicit protective mucosal and adaptive immune responses against Helicobacter pylori infection
Source: Front Immunol. 2025 Nov 4;16:1697591. doi: 10.3389/fimmu.2025.1697591 (PMC12623323; doi:10.3389/fimmu.2025.1697591)
Supplement: Supplementary file 1 [file DataSheet1.doc]

**Gram-positive enhancer matrix delivering FVpE via M cell targeting elicit protective mucosal and adaptive immune responses against *Helicobacter pylori* infection**

Furui Zhang^1, †^, Yaqin He^2, †^, Hongpeng Liu^1, †^, Jing Wu^2^, Xin Li^3^, Jiale Chen^2^, Linhan Ni^4^, Zhen Zhang^5^, Juan Chen^6, *^, Kunmei Liu^7, *^, Le Guo^2, *^

1 School of Laboratory, Ningxia Medical University, Yinchuan, China.

2 Ningxia Key Laboratory of Clinical and Pathogenic Microbiology, General Hospital of Ningxia Medical University (the First Clinical Medical College of Ningxia Medical University), Yinchuan, China.

3 Laboratory, Xi'an Hospital of Traditional Chinese Medicine, Xi'an, China.

4 College of Pharmacy, Ningxia Medical University, Yinchuan, China.

5 Department of Geriatrics and Special Needs Medicine, General Hospital of Ningxia Medical University, Yinchuan, China.

6 Department of Pulmonary and Critical Care Medcine, General Hospital of Ningxia Medical University, Yinchuan, China.

7 Ningxia Key Laboratory of Cerebrocranial Diseases, Ningxia Medical University, Yinchuan, China.

^†^ These authors made equal contributions: Furui Zhang, Yaqin He and Hongpeng Liu

^*^ Correspondence: Le Guo, Kunmei Liu and Juan Chen

Email: [guoletian@163.com](mailto:guoletian@163.com), lkm198507@126.com and [chenjuan7419@163.com](mailto:chenjuan7419@163.com)

**Table S**1 Primer sequences

| **Species** | **Name** | **Primer sequences（5’-3’）** |
| --- | --- | --- |
| *H. pylori* | *16S rRNA* | Forward: CTC ATT GCG AAG GCG ACC T |
|  |  | Reverse: TCT AAT CCT GTT TGC TCC CCA |
|  | *18S rRNA* | Forward: GCA ATT ATT CCC CAT GAA CG |
|  |  | Forward: GCA ATT ATT CCC CAT GAA CG |

**Table S**2 Histological scoring criteria

| **Score** | **Histological criteria** |
| --- | --- |
| 0 | The epithelial layer structure is intact, with no obvious damage |
| 1 | The epithelial layer structure is disorganized, accompanied by slight deformation of epithelial cells. |
| 2 | The structure of the superficial lamina propria is disorganized or 1/3 of the glandular structure is missing. |
| 3 | The intermediate lamina propria is disorganized or 2/3 of the glandular structure is missing. |
| 4 | The lower lamina propria is eroded or more than 2/3 of the glandular structure is missing. |

**
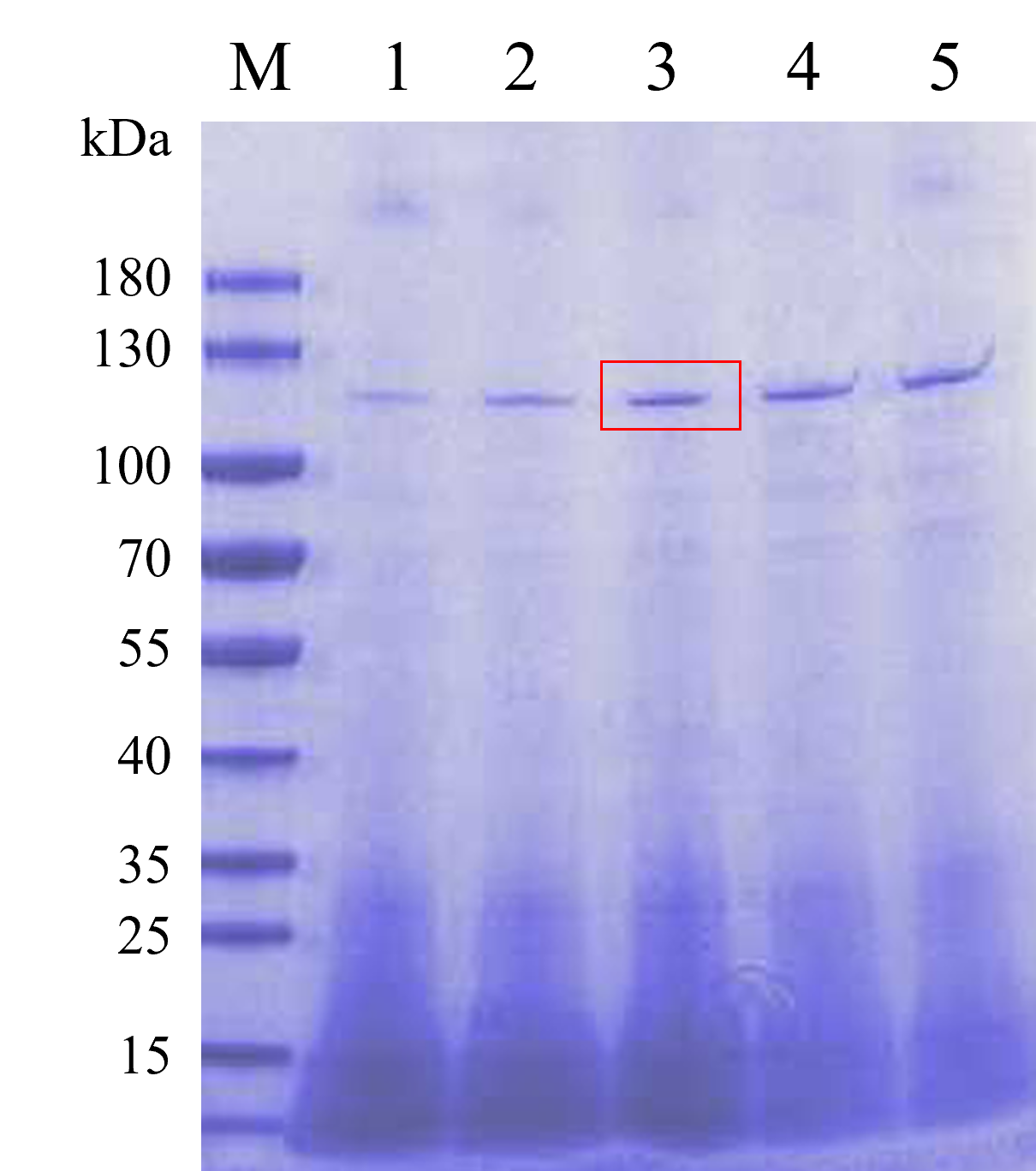
**

**Figure S1. SDS-PAGE analysis of the optimal binding ratio between GEM and SAM-FVpE antigen.** To determine the optimal binding ratio of GEM to SAM-FVpE, a constant amount of GEM (0.1 U, 1×10^7^ particles) was incubated with increasing amounts of SAM-FVpE (2.5, 5, 10, 20, and 40 μg). The lysate, containing the GEM-SAM-FVpE complexes, were collected and analyzed by SDS-PAGE. Lane 1: 0.1 U GEM + 2.5 μg SAM-FVpE, Lane 2: 0.1 U GEM + 5 μg SAM-FVpE, Lane 3: 0.1 U GEM + 10 μg SAM-FVpE, Lane 4: 0.1 U GEM + 20 μg SAM-FVpE, Lane 5: 0.1 U GEM + 40 μg SAM-FVpE.

**
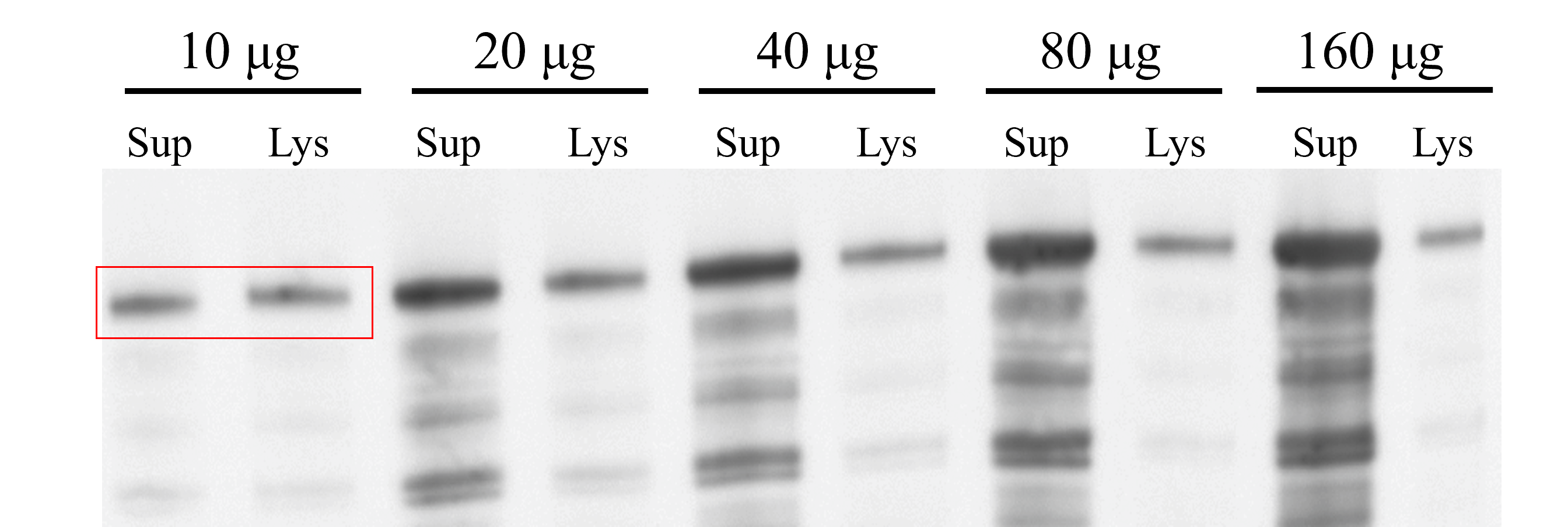
**

**Figure S2. Optimization of the binding conditions between GEM and SAM-FVpE antigen analyzed by Western blot.** To further determine the optimal binding ratio between GEM and SAM-FVpE while minimizing the consumption of purified protein, a constant amount of GEM (0.1 U, 1×10^7^ particles) was incubated with increasing amounts of SAM-FVpE (10, 20, 40, 80, and 160 μg). Both the supernatant (Sup) and the lysate (Lys) were collected and subjected to Western blot.
